# Physiological Perspectives on Molecular Mechanisms and Regulation of Vesicular Glutamate Transport: Lessons From Calyx of Held Synapses

**DOI:** 10.3389/fncel.2021.811892

**Published:** 2022-01-13

**Authors:** Tetsuya Hori, Shigeo Takamori

**Affiliations:** ^1^Cellular and Molecular Synaptic Function Unit, Okinawa Institute of Science and Technology Graduate University, Okinawa, Japan; ^2^Laboratory of Neural Membrane Biology, Graduate School of Brain Science, Doshisha University, Kyoto, Japan

**Keywords:** calyx of Held, glutamate, VGLUT, synaptic transmission, V-ATPase

## Abstract

Accumulation of glutamate, the primary excitatory neurotransmitter in the mammalian central nervous system, into presynaptic synaptic vesicles (SVs) depends upon three vesicular glutamate transporters (VGLUTs). Since VGLUTs are driven by a proton electrochemical gradient across the SV membrane generated by vacuolar-type H^+^-ATPases (V-ATPases), the rate of glutamate transport into SVs, as well as the amount of glutamate in SVs at equilibrium, are influenced by activities of both VGLUTs and V-ATPase. Despite emerging evidence that suggests various factors influencing glutamate transport by VGLUTs *in vitro*, little has been reported in physiological or pathological contexts to date. Historically, this was partially due to a lack of appropriate methods to monitor glutamate loading into SVs in living synapses. Furthermore, whether or not glutamate refilling of SVs can be rate-limiting for synaptic transmission is not well understood, primarily due to a lack of knowledge concerning the time required for vesicle reuse and refilling during repetitive stimulation. In this review, we first introduce a unique electrophysiological method to monitor glutamate refilling by VGLUTs in a giant model synapse from the calyx of Held in rodent brainstem slices, and we discuss the advantages and limitations of the method. We then introduce the current understanding of factors that potentially alter the amount and rate of glutamate refilling of SVs in this synapse, and discuss open questions from physiological viewpoints.

## Introduction

Upon the arrival of action potentials at presynaptic sites, synaptic vesicles (SVs) that store neurotransmitters in the lumen undergo exocytic fusion with the presynaptic plasma membrane, thereby releasing their contents to neighboring neurons. The main excitatory neurotransmitter in the mammalian brain is the acidic amino acid, glutamate. Three vesicular glutamate transporters (VGLUT1–3) are responsible for packaging it into SVs (Takamori, 2006). Deletion of each VGLUT gene in mice, as well as other model organisms such as *Drosophila*, largely silenced glutamatergic transmission, indicating that VGLUTs are essential for brain functions (Fremeau et al., 2004; Wojcik et al., 2004; Daniels et al., 2006). Furthermore, alterations of VGLUT expression, in addition to the expression of plasma membrane glutamate transporters (O’Donovan et al., 2017), are associated with a wide range of neurological disorders, such as epilepsy, anxiety and mood disorders, Alzheimer’s disease, Parkinson’s disease, and schizophrenia (summarized in a recent review by Pietrancosta et al., 2020). Therefore, mechanisms and regulators of vesicular glutamate transport mediated by VGLUTs offer potential treatment targets for these disorders.

**Figure 1:**
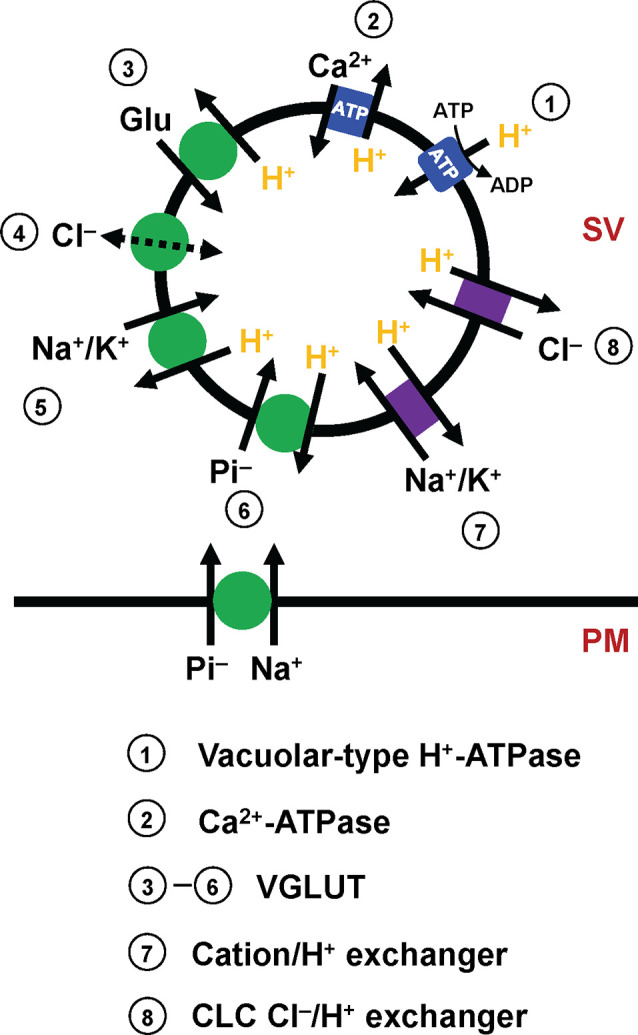
Molecular elements that affect glutamate transport into SVs. Mammalian SVs contain two primary pumps (blue square), vacuolar-type H^+^-ATPases (V-ATPases, 1) and plasma membrane Ca^2+^-ATPases (PMCAs, 2). V-ATPases create a proton electrochemical gradient ΔμH^+^, which is essential for glutamate transport by VGLUTs. PMCAs attenuate ΔpH, and conversely potentiate ΔΨ, thereby affecting VGLUT activity. The primary function of VGLUTs (3–6, green circle) is to transport glutamate into SVs, although how VGLUTs utilize ΔμH^+^ during the transport cycle has long been debated (3). In addition to glutamate transport, biochemical studies indicate that VGLUTs mediate various transport processes, such as proton-dependent Cl^−^ conductance (4), cation/proton exchange (5), and inorganic Pi transport (6). Importantly, VGLUTs are thought to mediate both Na^+^/Pi transport and H^+^/Pi, depending on locations. Although molecular identities are uncertain, SVs contain cation/H^+^ exchangers (7) and CLC-family members that mediate Cl^−^/H^+^ exchange (8), activities of which modulate the driving force for glutamate transport.

Like other neurotransmitters, glutamate transport into SVs is driven by a proton electrochemical gradient (ΔμH^+^) across SV membranes (Figure 1). The ΔμH^+^ is composed of both electrical (ΔΨ) and chemical (ΔpH) gradients, the balance of which is influenced by the presence of permeable ions (Takamori, 2016; Farsi et al., 2017). Biochemical analysis indicates that ΔΨ constitutes the dominant driver of glutamate transport (Maycox et al., 1988), although contributions of ΔpH or luminal pH have been a matter of intensive debate (Tabb et al., 1992; Schenck et al., 2009; Juge et al., 2010; Eriksen et al., 2016). Proportions of the two components of ΔμH^+^, as well as the net ΔμH^+^, are largely affected by permeant Cl^−^ ions. For instance, extravesicular (cytoplasmic) Cl^−^ can serve as a shunt for H^+^ movement, facilitating net H^+^ movement. As a result, external Cl^−^ ions increase ΔpH, while decreasing ΔΨ, as evidenced by *in vitro* experiments using isolated vesicles (Cidon and Sihra, 1989; Xie et al., 1989). Additionally, in living synapses, SVs are regenerated either directly from the plasma membrane or from endosome-like vacuoles derived from the plasma membrane (Gan and Watanabe, 2018). As a plausible consequence, newly regenerated SVs must contain an extracellular solution with high Cl^−^ concentrations (e.g., 130 mM), and Cl^−^ efflux will thus contribute to increase ΔΨ until the Cl^−^ gradient across the SV membrane reaches equilibrium. Such changes in the driving force by Cl^−^ would significantly modulate glutamate transport. Intriguingly, some observations indicate that Cl^−^ ions bind directly to VGLUT and allosterically modulate its activity (Hartinger and Jahn, 1993; Juge et al., 2010). Furthermore, accumulating evidence suggests that VGLUT itself exhibits Cl^−^ conductance (Bellocchio et al., 2000; Schenck et al., 2009), which is activated by H^+^ and eventually inhibits glutamate transport by a competing transport pathway (Eriksen et al., 2016) or increases it by compensating charge imbalance through an exchange mechanism (Schenck et al., 2009).

In addition to Cl^−^ conductance, recent *in vitro* experiments indicate that VGLUTs transport surprisingly diverse substances. One of these is inorganic phosphate. In fact, before the recognition of glutamate transport activities, VGLUT1 and VGLUT2 were originally cloned as plasma membrane Na^+^-dependent inorganic phosphate transporters, given that their heterologous expression stimulates Na^+^/Pi co-transport into *Xenopus* oocytes (Ni et al., 1994; Aihara et al., 2000; Bellocchio et al., 2000; Takamori et al., 2000). Recent results from VGLUT reconstitution not only support Na^+^-dependent phosphate transport (Juge et al., 2006), but indicate that VGLUTs also promote H^+^-dependent Pi transport into SVs, which competes with glutamate transport (Preobraschenski et al., 2018), indicating that VGLUTs transport Pi by utilizing two discrete driving forces, depending on their locations (Preobraschenski et al., 2018; Cheret et al., 2021). More surprisingly, VGLUTs also seem to mediate cation/H^+^ exchange in SVs, which would convert ΔpH to ΔΨ, thereby facilitating the ΔΨ-driven glutamate transport (Preobraschenski et al., 2014).

Despite accumulating *in vitro* evidence from mechanistic insights into glutamate transport modulation by various ions, the physiological relevance of the foregoing biochemical observations in living synapses is largely unexplored, mainly due to technical constraints in manipulating and quantitatively measuring glutamate and various ion concentrations in the cytoplasm and in vesicle lumens. Recent years have witnessed some important observations concerning vesicular glutamate transport mechanisms by utilizing a giant synapse—the calyx of Held synapse—as a model. Furthermore, a recent analysis of VGLUT1-deficient calyces has invoked several regulatory mechanisms regarding how VGLUT expression level, as well as expression of individual VGLUT isoforms, would impact synaptic transmission (Nakakubo et al., 2020). In this review, we will summarize key observations using the calyx of Held synapses that have shed further light on mechanisms and regulation of glutamate transport into SVs and will highlight some of the unknowns underlying the process.

## The Calyx of Held Synapse: A Model Synapse Suitable for Investigating Presynaptic Mechanisms

**Figure 2:**
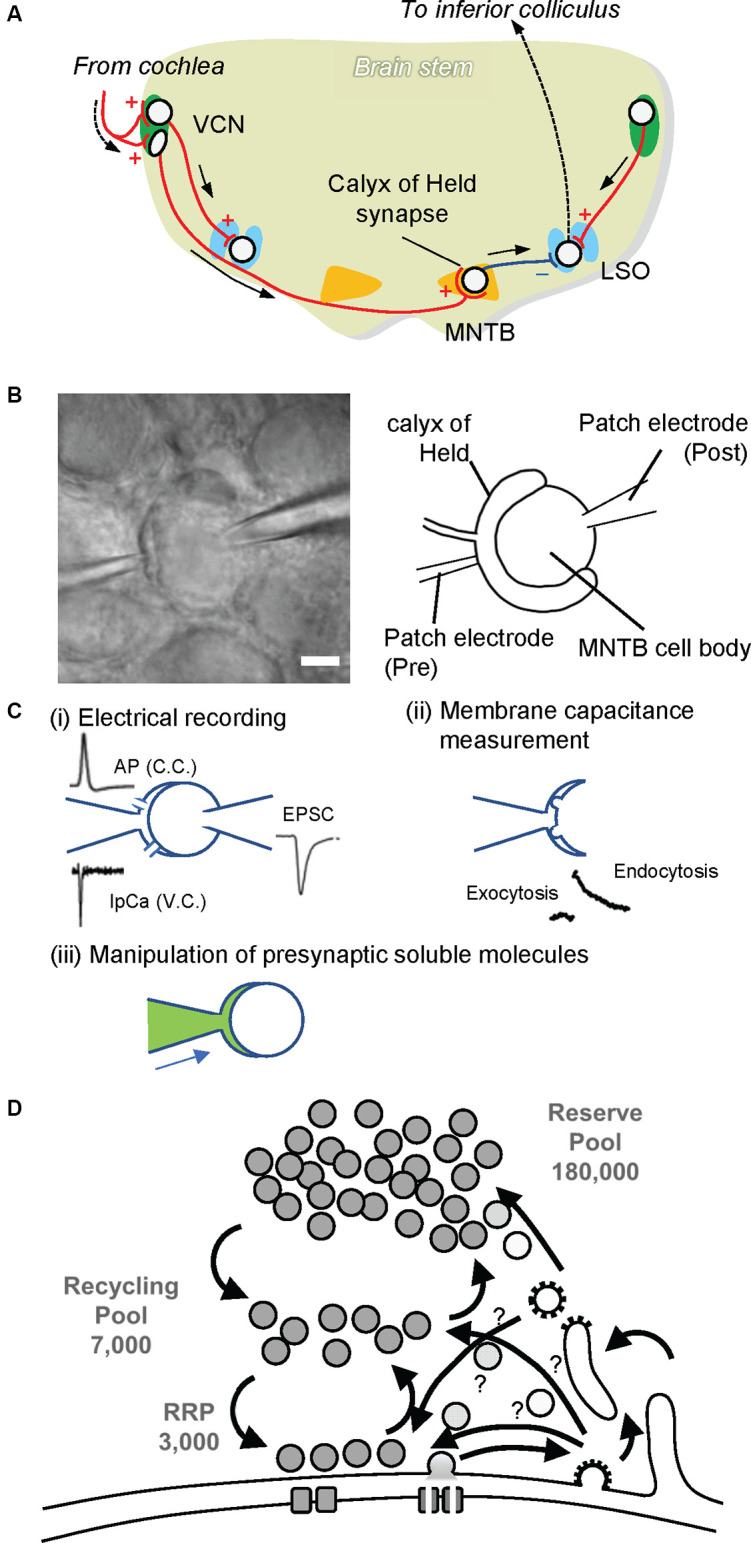
The calyx of Held synapse. **(A)** The auditory circuit in rodent brainstem. Globular and spherical bushy cells in the ventral cochlear nucleus (VCN) receive excitatory synaptic input from the cochlea. Spherical bushy cells send their axons to the ipsilateral lateral superior olive (LSO), whereas globular bushy cells project to the contralateral medial nucleus of the trapezoid body (MNTB). Excitatory synapses on MNTB principal cells, called the calyx of Held. MNTB principal cells form inhibitory synapses to the ipsilateral LSO. The LSO projects to the inferior colliculus, where sound information is integrated. Red lines with + and a blue line with — indicate excitatory and inhibitory inputs, respectively. **(B)** Paired whole-cell patch clamp recording at the calyx of Held. A patch electrode on the left is set to perform whole-cell patch clamp recording from the calyx of Held presynaptic terminal (Pre), while a patch electrode on the right is placed for electrical recordings whole-cellfrom MNTB principal cells (Post). The scale bar indicates 5 μm. **(C)** Various advantages of the presynaptic terminal patch clamp method. (i) By establishing paired patch clamping at the calyx of Held synapse, one can record action potentials (APs) under current clamp mode (C.C.) from presynaptic terminals, and Ca^2+^ currents (IpCa) under voltage clamp mode (V.C.). Simultaneously, excitatory postsynaptic currents (EPSCs) can be recorded from postsynaptic cells. In addition, through the electrode at the presynaptic terminal, one can trigger neurotransmitter release by applying depolarization pulses as well as local Ca^2+^ uncaging. (ii) SV exocytosis and subsequent membrane retrieval (endocytosis) can be monitored by membrane capacitance measurements. (iii) Through the presynaptic electrode, one can manipulate the composition of presynaptic cytoplasm by dialyzing it with an intra-pipet solution of the desired composition (green). This in turn enables the application of membrane-impermeable drugs directly into presynaptic terminals, washout of intrinsic glutamate from the cytoplasm, and the subsequent glutamate uncaging experiment to monitor glutamate refilling into emptied SVs. **(D)** A schematic drawing depicting three pools of SVs. A calyx terminal contains ~200,000 SVs, consisting of only ~3,000 synapses in the readily releasable pool (RRP) that swiftly responds upon AP arrival, ~7,000 synapses in the recycling pool that replenishes the RRP during sustained stimulation, and a reserve pool that participates only during intensive repetitive stimulation (Rizzoli and Betz, 2005).

The calyx of Held is the largest nerve terminal in the mammalian central nervous system, occupying 25–50% of the postsynaptic cell body, located in the medial nucleus of the trapezoid body (MNTB) within the superior olivary complex (Figure 2A). The origin of the calyx nerve terminal comes from the globular bushy cell, located in the ventral cochlear nucleus (VCN). Globular bushy cells are contacted by multiple large endings of auditory nerve fibers. The ability of bushy cells to encode temporal fine structure in the incident acoustic wave and their involvement in brainstem auditory circuits that mediate sound localization implicates the calyx of Held synapses in localizing sound in space.

During embryogenesis, multiple axons form synaptic contacts on neurons in the MNTB at around embryonic day 17, when synapses already have the ability to induce action potentials on the postsynaptic cell (Hoffpauir et al., 2010). Characteristic calyx presynaptic terminals emerge between postnatal days 2 and 4. During this period, only one major projection input remains, while other input fibers are eliminated, establishing a 1:1 correspondence between a calyx presynaptic terminal and the postsynaptic cell (Hoffpauir et al., 2006). Ear canals generally open at around postnatal day 10 in rodents. At this time, robust morphological and molecular changes occur in the calyx of Held synapses. Morphologically, the shape of the calyx of Held presynaptic termini changes from spoon-shaped to finger-shaped (Kandler and Friauf, 1993; Ford et al., 2009). In addition, the expression of various proteins related to synaptic transmission, including voltage-gated channels, SV proteins, and postsynaptic receptors drastically changes during hearing onset (Iwasaki and Takahashi, 1998; Futai et al., 2001; Blaesse et al., 2005). These expression changes underlie developmental changes in synaptic functions in this synapse (Iwasaki and Takahashi, 2001; Schneggenburger and Forsythe, 2006; Borst and Soria van Hoeve, 2012).

Structural analysis using electron microscopy revealed that calyx terminals are filled with spherical synaptic vesicles, an indication of excitatory connections, and they contain multiple active zones, a hallmark of presynaptic release sites. The number of synaptic sites ranges from 300 in mice to 600 in rats. Later studies showed that calyces contain ~180,000 synaptic vesicles per terminal (Satzler et al., 2002; de Lange et al., 2003; Neher, 2010). In accordance with the contemporary three-vesicle pool model (Rizzoli and Betz, 2005), the calyx of Held synapses contains ~3,000 vesicles in the readily releasable pool (RRP), which can be released immediately upon AP arrival (Sakaba and Neher, 2001). They also contain ~40,000 vesicles in the recycling pool, which replenish the RRP during sustained stimulation (de Lange et al., 2003; Yamashita et al., 2005), and ~180,000 vesicles in the reserve pool, which are used only during intense stimulation (Satzler et al., 2002; de Lange et al., 2003; Figure 2D). In spite of this extraordinary large number of total SVs, the sizes of each SV pool per release site are comparable to those of conventional synapses, e.g., small hippocampal synapses, making it a suitable model for glutamatergic presynaptic terminals (Rizzoli and Betz, 2005). Like other conventional synapses, efficient endocytic retrieval of SV membranes after exocytosis is essential to sustain transmission in this synapse (Yamashita et al., 2005), although modes of endocytosis, as well as the fate of endocytosed vesicles, i.e., which routes endocytosed materials travel until they are reused for exocytosis, and how long it takes, are not fully understood (Neher, 2010).

Because of its extraordinarily large size, the calyx of Held synapses in acutely prepared brain slices are amenable to patch clamp techniques (Figure 2B). Since the pioneering work by Forsythe (1994) showed that direct patch-clamp recording from the presynaptic plasma membrane is possible, calyx of Held synapses have been one of the most powerful preparations to investigate biophysical properties and underlying presynaptic molecular mechanisms of synaptic transmission in the mammalian brain. There are several reasons, to mention a few, that make the calyx of Held a suitable model for analysis. (1) It is a unique “one calyx–one MNTB” synapse. Although an MNTB neuron receives other inputs, mainly from surrounding interneurons, one bushy cell makes synaptic contact exclusively with one MNTB neuron. This simple connection ensures the identity of the origin of the postsynaptic response. It also ensures that spontaneous responses elicited by single-vesicle exocytosis and evoked responses elicited either by depolarization of the presynaptic membrane or by electrical stimulation of an afferent fiber are of the same origin. (2) It is a pure glutamatergic neuron, so that knowledge from the calyx of Held may be applicable to most other excitatory synapses throughout the brain. It should be noted, however, that unlike other glutamatergic synapses that predominantly express one of the two VGLUT isoforms (VGLUT1 and VGLUT2) in adulthood (Fremeau et al., 2001; Fujiyama et al., 2001), the calyx of Held synapse expresses both VGLUT1 and VGLUT2 at relatively high levels (Billups, 2005; Blaesse et al., 2005). Unlike hippocampus and neocortex in which VGLUT2 is weakly expressed in early development and is replaced by VGLUT1 during the 2nd or 3rd postnatal week (Fremeau et al., 2004; De Gois et al., 2005), VGLUT2 expression is constant until the 4th week while VGLUT1 expression gradually increases during this developmental stage (Billups, 2005). (3) Thanks to its extraordinarily large size, whole-cell patch clamping can easily be established at the presynaptic site. By doing so, one can directly record the presynaptic membrane potential in current clamp mode and activities of ion channels present on the presynaptic membrane in the form of currents in membranes under a voltage clamp configuration with extremely high spatio-temporal precision (Figure 2C). In particular, the Na^+^ current upon generation of an action potential (AP), the K^+^ current related to AP termination, and the Ca^2+^ current that is coupled to trigger SV exocytosis can be measured from the presynaptic plasma membrane. In addition, with membrane capacitance measurements, one can monitor activity-dependent SV dynamics, consisting of exocytic increases of membrane capacitance and subsequent decay due to compensatory endocytosis of exocytosed SV membranes (Sun and Wu, 2001). (4) Whole-cell presynaptic patch clamping enables experimental manipulations of presynaptic cytoplasmic composition, which cannot be achieved in conventional smaller synapses, i.e., dialysis of presynaptic cytoplasm with an intra-pipet solution, application of membrane-impermeable drugs into presynaptic cytoplasm through a pipet with defined concentrations, and so on. The inevitable downside of this manipulation is, however, that whole-cell patch clamping definitely leads to the loss of endogenous soluble molecules from presynaptic terminals, which may change the properties of presynaptic terminals in a physiological environment.

In addition to electrophysiologically amenable preparations of the calyx of Held synapses in acute brainstem slices, these giant presynaptic terminals were successfully reconstituted by culturing two types of dissociated cells derived from cochlear nuclei and from medial nuclei of the trapezoid body in the same dish (Dimitrov et al., 2016). This novel preparation allows genetic manipulation and enables them to be adapted for optical measurements of SV dynamics with simultaneous presynaptic electrical recordings, which cannot be readily achieved with acute slice preparations. However, the current protocol seems to hamper feasible applications due to a relatively low success rate, the necessity of long-term culture, e.g., DIV20–22, to establish single input-output pairs, and relatively small postsynaptic currents seen in this preparation (<1 nA), necessitating further optimization.

## Manipulation of Vesicular Glutamate Content by Dialyzing Presynaptic Terminals with Solutions Containing Various Glutamate Concentrations

Direct access to the giant presynaptic terminal of the calyx of Held using glass pipets allows us to clamp the presynaptic cytoplasm with a solution of the desired composition. In particular, one can clamp presynaptic glutamate concentrations and monitor vesicular glutamate contents through excitatory postsynaptic current (EPSC) recordings in a voltage-clamp configuration at postsynaptic MNTB cells. An additional *tour-de-force* technique to “micro-inject’ a desired solution through a thinner tube installed in a presynaptic patch pipet and connected to a syringe enables the consecutive exchange of presynaptic solutions. Switching a solution containing 10 mM glutamate to a glutamate-free solution results in a gradual decrease in both evoked EPSCs amplitudes and miniature EPSC amplitudes, albeit to a lesser extent, over 30 min, indicating that vesicular glutamate content can be depleted by the exocytic release of pre-filled glutamate and subsequent blockade of glutamate refilling of endocytosed vesicles (Ishikawa et al., 2002). The leakage of glutamate from pre-filled SVs does not seem to be a source of rundown (Ikeda and Bekkers, 2009; Takami et al., 2017). In turn, switching a solution with 1 mM glutamate to that with 100 mM glutamate increases both evoked and miniature EPSC amplitudes by ~100% and by ~50% respectively, indicating that vesicular glutamate content is critically determined by cytoplasmic glutamate concentrations, as previously indicated by a number of biochemical transport studies using isolated vesicles (Naito and Ueda, 1985; Wolosker et al., 1996; Wilson et al., 2005). Detailed assessment of cytoplasmic glutamate concentrations that are needed to maintain mEPSC amplitudes in an invasive situation (without presynaptic whole-cell recordings) revealed that the presynaptic glutamate concentration at the calyx of Held is ~1 mM (Ishikawa et al., 2002), which is within the rage of Kms of glutamate transport measured in isolated SVs. However, the seemingly non-saturable nature of glutamate contents in the presence of up to 100 mM is surprising and incompatible with biochemical transport assays *in vitro* (Naito and Ueda, 1985; Wolosker et al., 1996; Wilson et al., 2005). Thus, different mechanisms to regulate glutamate content at equilibrium *in vivo* must exist, e.g., changes in vesicle volume and in glutamate leakage that are associated with the exceeded glutamate refilling under these conditions that may be non-physiological.

In addition to the increase in quantal size in the presence of 100 mM [Glu]_cyto_, quantal content was concomitantly increased (Ishikawa et al., 2002), suggesting an increase either in the number of releasable vesicles or in the release probability of those vesicles, or a combination of both. Interestingly, experiments conducted later using hippocampal autaptic cultures support the concept that the degree of filling of SVs with glutamate affects their release probability, i.e., more glutamate in the vesicle increases the release probability (Herman et al., 2014). However, how the filling status of SVs with glutamate influences the release properties of SVs is unknown. It has been proposed that physical changes of the vesicle membrane due to the hyper-osmotic condition conferred by glutamate may be responsible. Likewise, the absence of glutamate is thought to cause the distorted morphology of empty SVs observed in VGLUT1-KO neurons under certain fixation conditions (Siksou et al., 2013; Herman et al., 2014). Interestingly, glutamate loading into isolated SVs induces an expansion of vesicle volume by ~100% *in vitro*, in which multi-transmembrane protein, Synaptic Vesicle-associated Glycoprotein 2 (SV2), performs a critical function (Budzinski et al., 2009). These results collectively indicate that the amount of glutamate in the lumen indirectly regulates the vesicle release probability *via* changes in the biophysical properties of SV membranes. Other potential molecular mechanisms controlling release probability conferred by distinct VGLUT-isoforms will be discussed below.

## Kinetics of Glutamate Transport into SVs at Calyx of Held Synapses

**Figure 3:**
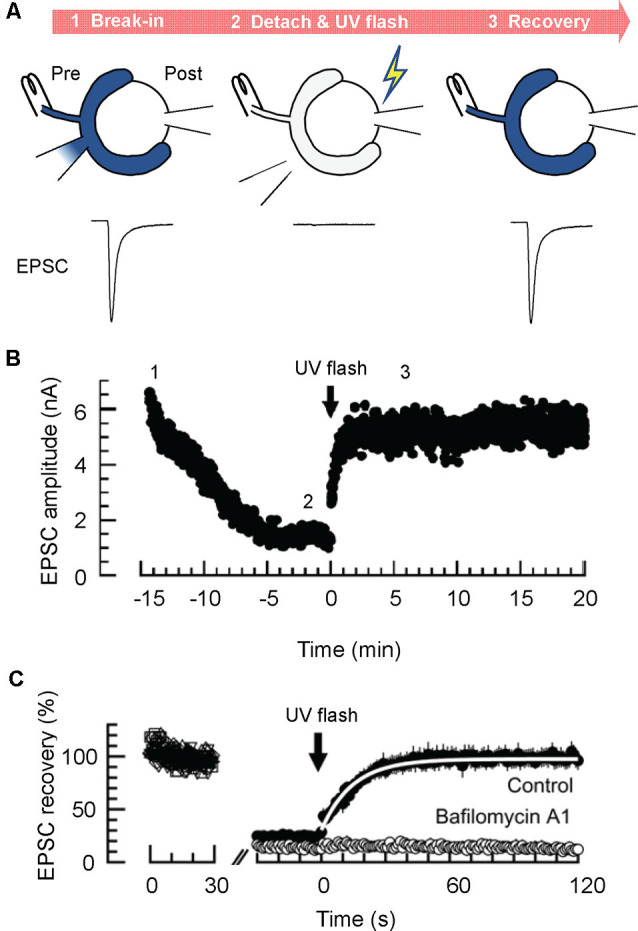
Glutamate uncaging experiments enable estimation of the rate of glutamate refilling into SVs in calyx of Held synapses. **(A)** Glutamate uncaging experiments. Dialysis of the presynaptic terminal with a glutamate-free solution containing 4-methoxy-7-nitroindolinyl (MNI)-caged glutamate is established, while an afferent fiber is continuously stimulated with a bipolar stimulation electrode (at 1 Hz; STEP1). After EPSCs decline substantially, the presynaptic patch pipet is detached, and a UV flash (1 s) is applied to induce a rapid increase of cytosolic glutamate by uncaging MNI-caged glutamate (STEP 2). This results in the recovery of EPSCs, which may reflect the rate of glutamate transport into pre-acidified, but empty SVs (STEP 3). **(B)** A representative time course of EPSCs monitored during glutamate uncaging experiments. **(C)** Representative magnified EPSC traces during glutamate uncaging experiments. Note that EPSCs recover nearly completely after glutamate uncaging under control conditions, whereas they do not recover in the presence of the V-ATPase blocker, bafilomycin A1. A white line indicates an exponential fit to the EPSCs to deduce the glutamate refilling rate. Panels in **(B,C)** are reproduced and slightly modified from Hori and Takahashi (2012).

Micro-perfusion of presynaptic terminals with solutions containing fixed glutamate concentrations described above, shed light on various essential points concerning vesicular glutamate content and its regulation in physiological contexts. However, since it is intrinsically difficult to measure the time required to replace presynaptic solutions using microinjection, the source of changes in EPSCs upon solution exchange cannot be identified with certainty, either due to the slow changes in steady-state glutamate content of pre-filled vesicles, or to changes in glutamate refilling into endocytosed SVs during measurements. To directly monitor dynamics of glutamate refilling into endocytosed “empty” SVs, Hori and Takahashi developed an efficient method to swiftly increase cytoplasmic glutamate concentrations using glutamate uncaging after vesicular glutamate was largely washed out by dialyzing the terminals with a glutamate-free solution (Hori and Takahashi, 2012). When simultaneous presynaptic and postsynaptic whole-cell recordings were made with a presynaptic pipet containing 0 mM glutamate and 10 mM 4-methoxy-7-nitroindolinyl (MNI)-glutamate, EPSCs elicited at 1 Hz declined gradually over ~15 min, due to depletion of the recycling pool (Figure 3). When the EPSC amplitude reached a low level, a UV flash (1 s) was applied to photorelease glutamate from MNI-glutamate in the presynaptic terminals (The presynaptic pipet was “detached’ from the terminals to avoid a rapid diffusion of released glutamate back into the pipet, and an afferent fiber was continuously stimulated to monitor EPSCs throughout measurement). Unlike replacement of solutions by microperfusion (from 1 mM to 100 mM glutamate), EPSCs recovered with much faster kinetics upon glutamate uncaging with a time constant of ~15 s, which is 10–100 times faster than those estimated in isolated vesicles (Naito and Ueda, 1985; Maycox et al., 1988; Carlson et al., 1989). Furthermore, titration of glutamate concentrations achieved by glutamate uncaging revealed that the Km of glutamate refilling measured in the calyces was 0.91 mM, which is in the range of those measured biochemically using isolated vesicles (Maycox et al., 1988; Carlson et al., 1989) as well as heterologous preparations expressing VGLUTs (Kaneko and Fujiyama, 2002). The large discrepancy in refilling kinetics between isolated vesicles and intact vesicles in the living nerve terminals is enigmatic but may arise from a loss of original luminal ionic composition, or from inevitable damage or loss of the VGLUT transport system during fractionation of vesicle membranes. Notably, SVs isolated from native brains by standard cell fractionation protocols lose glutamate completely during purification (Burger et al., 1989). Thus, it is conceivable that they also lose luminal ions that might influence glutamate transport.

Despite this first success toward estimating the rate of glutamate transport in living synapses, there remain several critical concerns regarding the protocol. First, as noted, uncaging MNI-caged glutamate produced unexpected cytotoxicity, which impede the recovery of EPSCs (Hori and Takahashi, 2012). Although the inclusion of 20 mM glutathione seemed to effectively prevent toxicity (on Ca^2+^ influx and exocytosis), the possibility of toxicity affecting VGLUT, V-ATPase, and other vesicular components inhibiting the glutamate transport rate cannot be fully excluded. Second, this protocol allows the measurement of glutamate transport into SVs that are already acidified by V-ATPase. If acidification of SVs is rate-limiting, glutamate refilling would take much longer as a whole. In fact, clathrin-coats by which newly-regenerated SVs are surrounded, severely inhibit ATP-dependent acidification of SVs (Farsi et al., 2018). Finally, cytoplasmic factors may have been removed after dialysis of presynaptic terminals, which potentially modulate the rate of glutamate transport into SVs (Ozkan et al., 1997; Winter et al., 2005).

## Unique Chloride Dependence of Vesicular Glutamate Transport

As summarized in the “Introduction” section, both external and luminal Cl^−^ concentrations have a profound influence on glutamate transport measured *in vitro*. In particular, external (cytoplasmic) Cl^−^ concentrations exhibit a biphasic effect on glutamate transport with maximal activity in the presence of 4–30 mM Cl^−^, depending on glutamate concentrations used for the transport assay (40 μM and 5 mM glutamate, respectively; Naito and Ueda, 1985; Wolosker et al., 1996). At calyx of Held synapses, Cl^−^ concentration at presynaptic terminals was ~21 mM (Price and Trussell, 2006), which seems to be optimal for glutamate transport measured *in vitro*. However, dialyzing presynaptic terminals with a solution containing various Cl^−^ concentrations from 5 to 100 mM did not cause any changes in mEPSC amplitudes (Price and Trussell, 2006), indicating that Cl^−^ concentrations do not affect steady-state glutamate content in SVs. This is compatible with biochemical observations using isolated SVs, in which external Cl^−^ concentrations affect steady-state glutamate content only when ΔpH is dissipated pharmacologically (Wolosker et al., 1996). On the contrary, the glutamate refilling rate monitored by glutamate uncaging experiments clearly shows similar biphasic dependence on cytosolic Cl^−^ concentrations with maximum glutamate transport at 30 mM (Hori and Takahashi, 2012). Further, effects of cytosolic Cl^−^ concentrations on the kinetics of glutamate transport and the magnitude of steady-state glutamate content, at least within the measured time frame, differ among Cl^−^ concentrations (Hori and Takahashi, 2012), indicating complex regulation mechanisms by Cl^−^, as suggested by biochemical analysis (Hartinger and Jahn, 1993; Wolosker et al., 1996). Importantly, synaptic fidelity during high-frequency stimulation, assessed by postsynaptic action potential generation, was retarded when presynaptic terminals were dialyzed with a solution having non-optimal presynaptic Cl^−^ concentrations (either 0.02 mM or 120 mM; Nakakubo et al., 2020). These observations at the calyx of Held synapses strengthen the contribution of cytosolic Cl^−^ in the regulation of glutamate transport. Since cytoplasmic Cl^−^ concentrations can be altered by activity of plasma membrane transporters, such as the K^+^-Cl^−^ cotransporter, KCC2, and a Na^+^-K^+^-2Cl^−^ cotransporter, NKCC1, during development, in general (Kaila et al., 2014) as well as upon synaptic inhibition that involves a transient Cl^−^ influx through GABA_A_ or glycine receptors expressed in calyx terminals (Turecek and Trussell, 2001, 2002; Trojanova et al., 2014), regulation of glutamate transport into SVs by Cl^−^ is likely to be physiologically relevant. Furthermore, changes in cytosolic [Cl^−^] are associated with various diseases such as epilepsy and chronic pain (Kaila et al., 2014), indicating its pathological implications.

In contrast to the effect of cytosolic [Cl^−^], contributions of luminal Cl^−^ on glutamate refilling in a physiological context remain to be determined. Although simple replacement of luminal Cl^−^ at living synapses can be achieved, in principle, by turnover of vesicle pools in the presence of external solution with desired Cl^−^ concentrations, the large size of vesicle pools in the calyx of Held may impede the feasibility of assessment by postsynaptic recordings. In cultured hippocampal neurons, however, it seems that glutamate loading is associated with Cl^−^ efflux, and luminal Cl^−^ is critical for efficient vesicle acidification, indicating pivotal roles of luminal Cl^−^ on glutamate refilling in living synapses (Martineau et al., 2017).

## Regulation of Vesicular Glutamate Content by Cytoplasmic Cations Through Cation/H^+^ Exchange Mechanisms on Svs

Biochemical analysis of isolated SVs revealed that SVs exhibit an electro-neutral cation/H^+^ exchange activity, which converts ΔpH to ΔΨ, thereby facilitating glutamate transport into SVs (Goh et al., 2011). Furthermore, reconstitution of VGLUTs suggested that VGLUT itself mediates the cation/H^+^ exchange activity (Preobraschenski et al., 2014). These observations raise the possibility that changes in presynaptic cation concentrations may regulate vesicular glutamate transport and have the potential to influence synaptic transmission.

Cytosolic Na^+^ concentration is maintained at relatively low levels (~15 mM) by the activity of Na^+^/K^+^-ATPases on the plasma membrane. At calyx of Held terminals, hyperpolarization-activated cyclin nucleotide-gated (HCN) channels, which allow Na^+^ to pass through the membrane, also contribute to the resting cytosolic Na^+^ concentration. Activation of HCN channels increases resting Na^+^ concentrations by ~5 mM (Huang and Trussell, 2014). Further, the Na^+^ influx through voltage-gated Na^+^ channels during repetitive AP firing also contributes to increased cytosolic [Na^+^]. In fact, [Na^+^] reaches ~80 mM when 100-Hz stimulation is applied for 10 s (Huang and Trussell, 2014). As such, presynaptic [Na^+^] is subjected to control at physiologically relevant conditions.

**Figure 4:**
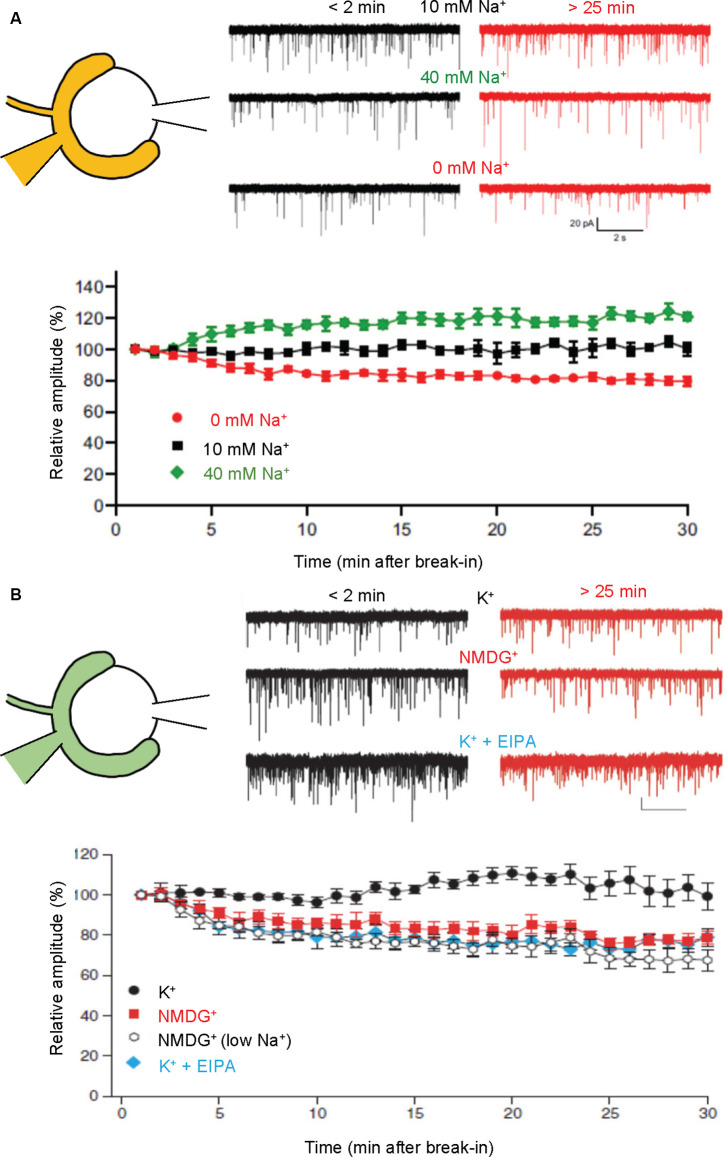
Presynaptic cations regulate mEPSC amplitudes through cation/H^+^ exchange in SVs. Paired recordings were performed from both pre- and postsynaptic compartments in a calyx of Held-MNTB neuron. mEPSCs were recorded postsynaptically immediately (within 2 min) and 25–30 min after a break-in to the presynaptic terminal with a pipet containing indicated buffers. Relative mEPSC amplitudes of initial mEPSCs were plotted as a function of time after a break-in. **(A)** Traces indicate mEPSCs measured under control (10 mM Na^+^; top), high Na^+^ (40 mM Na^+^, middle), and Na^+^-free (0 mM, bottom) immediately after break-in (<2 min, black) and after 25–30 min (>25 min, red). The bottom plot indicates mEPSC amplitudes relative to initial mEPSC amplitudes in the presence of 0 mM Na^+^ (red), 10 mM Na^+^ (black), and 40 mM Na^+^. Figures were modified from Huang and Trussell (2014). **(B)** Traces indicate mEPSCs measured under control (130 mM K^+^; top), K^+^-free (130 mM NMDG^+^; middle), and 130 mM K^+^ with EIPA (bottom) immediately after break-in (<2 min, black) and after 25–30 min (>25 min, red). The bottom plot indicates mEPSC amplitudes relative to initial mEPSCs in the presence of 130 mM K^+^ (K^+^; black circle), 130 mM NMDG^+^ (NMDG^+^; red), 10 mM NaCl, K^+^-free solution [NMDG^+^ (low Na^+^); open], and 130 mM K^+^ with 50–100 μM EIPA (K^+^ + EIPA; blue). Figures were modified from Goh et al. (2011).

Dialysis of calyces with a buffer containing high Na^+^ (40 mM) significantly increases both amplitude and frequency of mEPSCs, whereas that with a buffer lacking Na^+^ (0 mM) decreases them (Huang and Trussell, 2014; Figure 4A). Furthermore, pharmacological activation of HCN channels through cAMP activation increases mEPSC amplitudes, while inhibition of HCN channels decreases mEPSC amplitudes (Huang and Trussell, 2014). All these observations are compatible with a proposal from biochemical transport assays, that activation of Na^+^/H^+^ exchange potentiates ΔΨ, which would optimally drive glutamate transport.

Although regulation of presynaptic [K^+^] is less understood, manipulations of cytoplasmic [K^+^] at the calyx of Held synapses exerted similar effects on miniature EPSC amplitudes, i.e., complete replacement of presynaptic K^+^ with NMDG^+^ resulted in a gradual decline of mEPSC amplitudes by ~30%, while no decrease was observed in the presence of 130 mM K^+^ (Figure 4B; Goh et al., 2011). Interestingly, the inclusion of 10 mM Na^+^ in a pipet solution, which mimics physiological conditions to some extent, did not reverse the reduction of mEPSC amplitudes in the absence of K^+^, supporting the importance of K^+^ rather than Na^+^ in maintaining vesicular glutamate content under resting conditions. Finally, EIPA, an inhibitor of the Na^+^/H^+^ exchanger, also resulted in the reduction of mEPSC amplitudes to an extent similar to that in the absence of K^+^, indicating that the Na^+^/H^+^ exchanger is involved in this regulation, consistent with biochemical results.

Despite clear indications that cation/H^+^ exchange activity on SVs regulates vesicular glutamate contents under physiological conditions, the molecular identity of the Na^+^/H^+^ exchanger on SVs, as well as its contribution to the intrinsic cation/H^+^ exchange activity in VGLUTs are still uncertain. Among the SLC9/sodium proton exchanger (NHE) family, some members of which are suggested to be responsible for regulating organellar acidity in various cell types and tissues (Donowitz et al., 2013), NHE-1, -6, and -7 were identified in isolated SV membranes by a recent proteomic study (Taoufiq et al., 2020), and NHE6 is enriched in SVs (Preobraschenski et al., 2014). Of note, recent evidence from hippocampal neurons suggests that NHE6 may be central because knock-down of NHE6 alone results in a reduction of mEPSC amplitudes (Lee et al., 2021a, b). Furthermore, secretory carrier membrane protein 5 (SCAMP5), one of the genuine SV residents (Takamori et al., 2006), is responsible for proper sorting of NHE6 to fusion-competent SVs, and SCAMP5 knock-down also results in a similar reduction in mEPSCs. Since both NHE6 and SCAMP5 are associated with autism spectrum disorder (ASDs; Morrow et al., 2008; Castermans et al., 2010; Kondapalli et al., 2014; Schwede et al., 2014), regulation of vesicular glutamate content by NHE6 and SCAMP5 may be implicated in the pathogenesis of ASDs. Whether the same mechanisms are implemented in the calyx of Held synapses needs further investigation.

## Is Vesicular Refilling A Rate-Limiting Step for Neurotransmission?

Given the rate of glutamate transport into SVs, an important question remains as to whether the refilling speed can be rate-limiting for glutamatergic transmission. This can happen if the reuse of vesicles that have undergone exocytosis is faster than the time required for complete vesicle refilling (<20 s). Although we currently do not know the time required for vesicle reuse, recent studies using hippocampal synapses as well as cerebellar mossy fiber terminals in slices (Watanabe et al., 2013, 2014; Delvendahl et al., 2016), indicate that at physiological temperature, clathrin-independent rapid endocytosis occurs much more rapidly than previously believed (<100 ms), leaving room for exocytosis of incompletely filled vesicles upon vesicle reuse.

A suggestion of rapid reuse of incompletely filled vesicles comes from experiments in which vesicular glutamate transport was abolished by dialyzing presynaptic terminals with a glutamate-free pipet solution, while EPSCs were continuously monitored (Hori and Takahashi, 2012). Immediately after whole-cell voltage clamp was established at a presynaptic terminal, EPSCs evoked by afferent fiber stimulation at 1 Hz tend to decline when the pipet contained glutamate-free solution, although systematic analysis has not been done to determine how long it takes to detect a significant decrease from the initial EPSCs. This rapid decline was not observed when the pipet solution contained 3 mM glutamate, strongly indicating that SVs that had experienced exocytosis and had lost glutamate were reused multiple times during this short time. Consistent with this observation, blockade of glutamate refilling either by the V-ATPase inhibitor, folimycin, or by attenuating ΔμH^+^ buildup of endocytosed vesicles with strong buffers, results in rapid synaptic depression in hippocampal preparations (Ertunc et al., 2007). Oddly, rundown observed in the presence of another membrane-permeable V-ATPase inhibitor, bafilomycin, did not seem to cause immediate depression with the same onset (Hori and Takahashi, 2012). This is probably due to a side effect of bafilomycin, by which the release probability of SVs dramatically increases, which would mask the initial synaptic rundown due to the blockade of glutamate transport (Ikeda and Bekkers, 2009).

**Figure 5:**
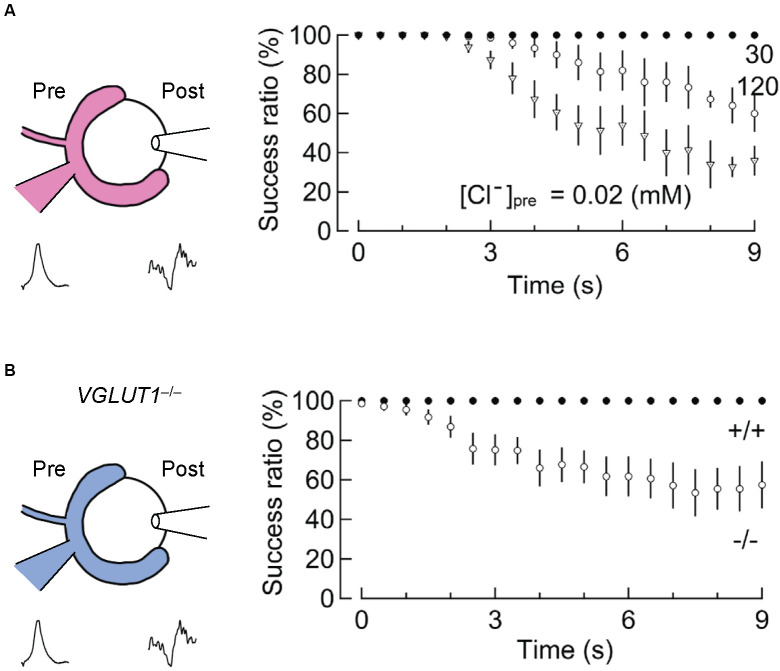
Slowed vesicular glutamate refilling impairs synaptic fidelity during high-frequency firing. **(A)** The impairment of synaptic fidelity when presynaptic Cl^−^ concentrations are not optimal for efficient glutamate refilling. In the presence of very low [Cl^−^]_pre_ (0.02 mM) or high [Cl^−^]_pre_ (120 mM), at which rates of glutamate refilling are dramatically slowed, synaptic fidelity upon long high-frequency stimulation (100 Hz) causes apparent synaptic failure within several seconds. This synaptic failure does not occur in the presence of 30 mM [Cl^−^]_pre_ within these time frames (modified from Nakakubo et al., 2020). **(B)** Impairment of synaptic fidelity in the calyx of Held synapses of *VGLUT1*^−/−^ mice. Despite considerable differences in basic synaptic parameters of the calyx of Held synapses between wild-type and *VGLUT1*^−/−^ mice, the rate of glutamate refilling in SVs is slowed in VGLUT1-decifient synapses by ~4-fold compared to wild-type. Presumably, as a consequence, synaptic fidelity during high-frequency firing is impaired within several seconds in VGLUT1-deficient synapses. Figures are modified from Nakakubo et al. (2020).

A second indication comes from an experiment in which the input-output relationship of presynaptic and postsynaptic AP firings is monitored, while vesicular glutamate transport is slowed by changing the presynaptic Cl^−^ concentrations (Nakakubo et al., 2020). Essentially, the calyx of Held synapses 16–19 days after birth endure a train of 1,000 stimuli at 100 Hz (for 10 s) without large failures (Figure 5A). However, when presynaptic Cl^−^ concentrations are clamped either at very low (0.02 mM) or at very high (120 mM) concentrations, both of which significantly retard glutamate refilling in this synapse (Hori and Takahashi, 2012), synaptic failures occur within 3–5 s (Nakakubo et al., 2020). This was also observed when VGLUT1 was genetically deleted (see below for details; Nakakubo et al., 2020; Figure 5B), supporting the notion that efficient vesicle refilling with glutamate can be rate-limiting for synaptic transmission during high-firing. Knowing that the rate of glutamate refilling measured under relatively milder conditions is much longer (τ ~15 s), this, in turn, suggests that mechanism(s) to accelerate vesicular refilling must exist during intensive stimulation. Whether Na^+^ or Ca^2+^, the latter of which also converts ΔpH to ΔΨ to facilitate glutamate uptake *in vitro* (Goncalves et al., 1999a, b; Ono et al., 2019), enhances glutamate refilling in these situations remains to be explored.

## Which Matters Most, Vglut Isoforms Or Total Vglut Expression at The Calyx of Held?

Expression of both VGLUT1 and VGLUT2 at the calyx of Held synapses (Billups, 2005) offers a unique opportunity to address possible functional differences between them using VGLUT1 knockout mice. While the transmembrane domains of the three VGLUTs are almost identical, N-termini and C-termini of VGLUTs, both of which face the cytoplasm, are quite different (Reimer, 2013). In fact, transport properties, i.e., the transport rate, the biphasic dependence on extra-vesicular [Cl^−^], the predominant utilization of ΔΨ, are quite similar among the three isoforms, despite some diversity in the requirement of ΔpH for transport (Bai et al., 2001; Kaneko and Fujiyama, 2002; Eriksen et al., 2016). In turn, distribution of these transporters in a heterologous system, as well as recycling properties and dynamics within presynaptic terminals, seem to differ among the isoforms in several respects (Voglmaier et al., 2006; Guillaud et al., 2017; Li et al., 2017), indicating differences in trafficking and sorting. Furthermore, in previous experiments using hippocampal autapses, as well as hippocampal slices, in which VGLUT1 expression predominates over other isoforms, it was reported that VGLUT1 confers low release probability, whereas VGLUT2 bestows high release probability (Weston et al., 2011; He et al., 2012). It is also unclear, in a subpopulation of hippocampal neurons as well as cortical neurons that express both VGLUT1 and VGLUT2, whether they are expressed at the same boutons (or even on the same vesicles) or whether they are segregated into distinct release sites (or distinct boutons; Fremeau et al., 2004; Schuske and Jorgensen, 2004; Wojcik et al., 2004; De Gois et al., 2005; Herzog et al., 2006). These studies also reached different conclusions as to how net VGLUT expression on an SV affects the quantal size of glutamate (Fremeau et al., 2004; Wojcik et al., 2004; Wilson et al., 2005; Herman et al., 2014). These complex issues have recently been addressed by analyzing the calyx of Held synapses derived from VGLUT1-KO mice (Nakakubo et al., 2020). It should be noted that since VGLUT1-KO mice die at around ~20 days after birth, most experiments were performed during postnatal days 16–19.

The first question is whether VGLUT1 and VGLUT2 are segregated into distinct vesicle populations. An immunohistochemical approach by triple staining of VGLUT1, VGLUT2, and an SV marker synaptophysin (Syp) revealed that although substantial populations of vesicle clusters are dominated by one of the two VGLUT isoforms, the majority of Syp-positive presynaptic structures contain both isoforms at different levels, consistent with other studies (Billups, 2005; Blaesse et al., 2005). Interestingly, unlike hippocampal preparations where VGLUT1 expression clearly predominates (Fremeau et al., 2004; Wojcik et al., 2004), there are no alterations in evoked EPSC amplitudes, or in the frequency and amplitude of miniature EPSCs in VGLUT1-deficient calyx of Held synapses (Nakakubo et al., 2020). Although exact copy numbers of both VGLUT isoforms in wild-type calyces and in VGLUT1-deficient calyces are difficult to measure, these data suggest that all “releasable” SVs in the calyx of Held synapses may contain at least a single copy of both VGLUT isoforms, and that VGLUT1-loss results in a decrease of net VGLUT expression of unknown extent (note that the average copy number of VGLUTs in an SV was estimated to be ~10; Takamori et al., 2006). This interpretation is compatible with studies of *Drosophila* neuromuscular junctions in which gradual reduction of the ortholog, DVGLUT, results in a reduction of mini frequencies, but not mini amplitudes, arguing that a single copy of VGLUT on an SV suffices to fill up SVs completely (Daniels et al., 2006). It also supports observations in rodent neurons that decreasing VGLUT3 expression in various mutants by as much as ~80% only minimally affects VGLUT3 function (Ramet et al., 2017). It seems, however, incompatible with observations that decreased VGLUT expression (VGLUT heterogyzous) resulted in decreased mEPSC amplitudes or increased mEPSC amplitudes when VGLUT is overexpressed (Wojcik et al., 2004; Wilson et al., 2005; Moechars et al., 2006, but see Fremeau et al., 2004). As it stands, with these contradictory observations, whether and how glutamate content in SVs is controlled by VGLUT levels remains controversial.

It seems conceivable, however, that VGLUT levels affect refilling speed until SVs are fully refilled with glutamate. When glutamate refilling after presynaptic glutamate washout is measured by glutamate uncaging, glutamate refilling was substantially slowed in VGLUT1-deficient synapses (τ ~80 s at room temperature), i.e., four times slower than observed in wild-type synapses (Nakakubo et al., 2020). In addition, recovery of EPSCs after glutamate uncaging was hardly observed when synapses were continuously stimulated at 1 Hz, necessitating lower stimulation at 0.1 Hz to observe the recovery of EPSCs in VGLUT1-deficient synapses. This was probably due to the involvement of exocytosis of partially re-filled SVs that could never reach a fully refilled state at continuous 1-Hz stimulation. The notion that VGLUT levels dictate the speed of refilling, but not the steady-state levels of glutamate content observed in the calyx of Held synapses is somewhat contradictory to earlier biochemical transport assays using pharmacological manipulations, arguing that the number of available VGLUTs on SVs influences the magnitude and to a lesser extent, the rate of glutamate uptake (Wilson et al., 2005), although the reason remains enigmatic.

Studies using hippocampal preparations revealed that EPSCs persisting in VGLUT1-KO neurons, apparently mediated by VGLUT2, exhibited higher release probability and rapid recovery after synaptic depression (Fremeau et al., 2004; Weston et al., 2011). Although these features seen in VGLUT1-deficient synapses have typically been attributed to VGLUT2, it is also possible that other components of respective synapses contribute to these different features. By taking advantage of the calyx of Held synapses in which both VGLUT1 and VGLUT2 are normally expressed on the same vesicles, albeit to different extents, analysis of the remaining EPSCs in VGLUT1-deficient calyces may provide deeper insights into differences in synaptic properties conferred by VGLUT isoforms. Estimation of RRP sizes and release probabilities deduced from short-term depression (STD) reveal that VGLUT2-laden vesicles exhibit a higher release probability than wild-type vesicles, while RRP sizes were not altered. Furthermore, recovery from STD was faster in VGLUT1-deficient synapses than in wild-type synapses, which was ablated in the presence of EGTA, indicating that faster replenishment of RRP by VGLUT2-laden vesicles is mediated by a Ca^2+^-dependent process. These observations are largely compatible with reports using hippocampal preparations from VGLUT1-KO mice (Fremeau et al., 2004), and strengthen the notion that expression of VGLUT isoforms regulates vesicle dynamics. The best-studied molecular difference among VGLUTs is the presence of a proline-rich domain at the carboxyl-terminal tail of VGLUT1, which offers a binding site for an endocytosis-related protein, endophilin (De Gois et al., 2006; Vinatier et al., 2006; Voglmaier et al., 2006). Although endophilin binding to VGLUT1 reportedly facilitates endocytosis of VGLUT1 during mild prolonged repetitive stimulation (Voglmaier et al., 2006) and reduces SV-release probability (Weston et al., 2011), a recent study using hippocampal neurons suggests that it also reduces SV mobility mediated by an additional endocytic protein, intersectin (Zhang et al., 2019), presumably representing a molecular mechanism underlying faster recovery after STD in the absence of VGLUT1. It should be noted, however, that vesicle tracking experiments on heterologous expression of fluorescently-labeled VGLUT1 and VGLUT2 in unique culture preparations of the calyx of Held terminals revealed that VGLUT1-laden vesicles move faster and travel longer distances than VGLUT2-laden vesicles (Guillaud et al., 2017), incompatible with facilitation of EPSC recovery from STD observed in VGLUT1-deficient synapses. How the binding ability of VGLUT1 to endophilin, which has been implicated in endocytosis, confers low release probability is difficult to explain. It was proposed that the number of available endophilin molecules influences vesicle release probability, and that VGLUT1 acts as an intrinsic “buffer” to inactivate endophilin by reducing its cytoplasmic concentrations (Weston et al., 2011). Intriguingly, more recent studies indicate that endophilin is involved directly in vesicle priming and fusion of neurosecretory granules in chromaffin cells and in modulation of presynaptic Ca^2+^ channel function in rodent cochlear inner hair cells (Kroll et al., 2019; Gowrisankaran et al., 2020), presumably relating to endophilin-dependent distinct release probabilities between VGLUT1- and VGLUT2-encoding synapses. Currently, precise actions of endophilin on the regulation of release probability and the presence of other key partners involved in these VGLUT isoform-specific properties in SV mobility and physiological consequences remain largely unknown.

## Concluding Remarks

In this review, we introduced recent key findings concerning mechanisms of vesicular glutamate transport processes in the calyx of Held and discussed their physiological relevance. Although mechanistic insights obtained from *in vitro* studies also suggest the importance of luminal ions that are engulfed by vesicles during endocytosis, e.g., facilitation of glutamate refilling by luminal Cl^−^ during the initial phase of glutamate loading, it is still uncertain how much this proposed mechanism contributes in physiological contexts. As for the refilling speed, studies using presynaptic glutamate uncaging indicate that it is much faster than rates observed biochemically using isolated SVs. Yet, with some experimental concerns and possible rapid use of filled vesicles during highly repetitive stimulation described above, it may be even faster than measured values. In addition to multiple modes of SV endocytosis with distinct time scales depending on stimulation strength, the time required for reuse of endocytosed vesicles may be an important key to fully understanding whether refilling speed can be rate-limiting for neurotransmission. A future innovation that enables direct observations of glutamate refilling of individual SVs at presynaptic terminals, in addition to indirect measures of synaptic outputs from postsynaptic cells, under precise manipulations and stimulation will likely give us the answer.

## Author Contributions

TH and ST: conceptualization, manuscript initial draft, manuscript critical correction, and approval of final version. All authors contributed to the article and approved the submitted version.

## Conflict of Interest

The authors declare that the research was conducted in the absence of any commercial or financial relationships that could be construed as a potential conflict of interest.

## Publisher’s Note

All claims expressed in this article are solely those of the authors and do not necessarily represent those of their affiliated organizations, or those of the publisher, the editors and the reviewers. Any product that may be evaluated in this article, or claim that may be made by its manufacturer, is not guaranteed or endorsed by the publisher.

## References

[B1] Aihara Y. Mashima H. Onda H. Hisano S. Kasuya H. Hori T.. (2000). Molecular cloning of a novel brain-type Na(+)-dependent inorganic phosphate cotransporter. J. Neurochem. 74, 2622–2625. 10.1046/j.1471-4159.2000.0742622.x 10820226

[B2] Bai L. Xu H. Collins J. F. Ghishan F. K. (2001). Molecular and functional analysis of a novel neuronal vesicular glutamate transporter. J. Biol. Chem. 276, 36764–36769. 10.1074/jbc.M104578200 11432869

[B3] Bellocchio E. E. Reimer R. J. Fremeau R. T. Jr. Edwards R. H. (2000). Uptake of glutamate into synaptic vesicles by an inorganic phosphate transporter. Science 289, 957–960. 10.1126/science.289.5481.957 10938000

[B4] Billups B. (2005). Colocalization of vesicular glutamate transporters in the rat superior olivary complex. Neurosci. Lett. 382, 66–70. 10.1016/j.neulet.2005.02.071 15911123

[B5] Blaesse P. Ehrhardt S. Friauf E. Nothwang H. G. (2005). Developmental pattern of three vesicular glutamate transporters in the rat superior olivary complex. Cell Tissue Res. 320, 33–50. 10.1007/s00441-004-1054-8 15714284

[B6] Borst J. G. Soria van Hoeve J. (2012). The calyx of held synapse: from model synapse to auditory relay. Annu. Rev. Physiol. 74, 199–224. 10.1146/annurev-physiol-020911-153236 22035348

[B150] Budzinski K. L. Allen R. W. Fujimoto B. S. Kensel-Hammes P. Belnap D. M. Bajjalieh S. M. (2009). Large structural change in isolated synaptic vesicles upon loading with neurotransmitter. Biophys J. 97, 2577–2584. 10.1016/j.bpj.2009.08.032 19883601 PMC2770603

[B7] Burger P. M. Mehl E. Cameron P. L. Maycox P. R. Baumert M. Lottspeich F.. (1989). Synaptic vesicles immunoisolated from rat cerebral cortex contain high levels of glutamate. Neuron 3, 715–720. 10.1016/0896-6273(89)90240-7 2577130

[B8] Carlson M. D. Kish P. E. Ueda T. (1989). Glutamate uptake into synaptic vesicles: competitive inhibition by bromocriptine. J. Neurochem. 53, 1889–1894. 10.1111/j.1471-4159.1989.tb09258.x 2809599

[B9] Castermans D. Volders K. Crepel A. Backx L. De Vos R. Freson K.. (2010). SCAMP5, NBEA and AMISYN: three candidate genes for autism involved in secretion of large dense-core vesicles. Hum. Mol. Genet. 19, 1368–1378. 10.1093/hmg/ddq013 20071347

[B10] Cheret C. Ganzella M. Preobraschenski J. Jahn R. Ahnert-Hilger G. (2021). Vesicular glutamate transporters (SLCA17 A6, 7, 8) control synaptic phosphate levels. Cell Rep. 34:108623. 10.1016/j.celrep.2020.108623 33440152 PMC7809625

[B11] Cidon S. Sihra T. S. (1989). Characterization of a H+-ATPase in rat brain synaptic vesicles. coupling to L-glutamate transport. J. Biol. Chem. 264, 8281–8288. 2566604

[B12] Daniels R. W. Collins C. A. Chen K. Gelfand M. V. Featherstone D. E. DiAntonio A. (2006). A single vesicular glutamate transporter is sufficient to fill a synaptic vesicle. Neuron 49, 11–16. 10.1016/j.neuron.2005.11.032 16387635 PMC2248602

[B13] De Gois S. Jeanclos E. Morris M. Grewal S. Varoqui H. Erickson J. D. (2006). Identification of endophilins 1 and 3 as selective binding partners for VGLUT1 and their co-localization in neocortical glutamatergic synapses: implications for vesicular glutamate transporter trafficking and excitatory vesicle formation. Cell Mol. Neurobiol. 26, 679–693. 10.1007/s10571-006-9054-8 16710756 PMC11520632

[B14] De Gois S. Schafer M. K. Defamie N. Chen C. Ricci A. Weihe E.. (2005). Homeostatic scaling of vesicular glutamate and GABA transporter expression in rat neocortical circuits. J. Neurosci. 25, 7121–7133. 10.1523/JNEUROSCI.5221-04.2005 16079394 PMC6725238

[B15] de Lange R. P. de Roos A. D. Borst J. G. (2003). Two modes of vesicle recycling in the rat calyx of Held. J. Neurosci. 23, 10164–10173. 10.1523/JNEUROSCI.23-31-10164.2003 14602833 PMC6740849

[B16] Delvendahl I. Vyleta N. P. von Gersdorff H. Hallermann S. (2016). Fast, temperature-sensitive and clathrin-independent endocytosis at central synapses. Neuron 90, 492–498. 10.1016/j.neuron.2016.03.013 27146271 PMC5125781

[B17] Dimitrov D. Takagi H. Guillaud L. Saitoh N. Eguchi K. Takahashi T. (2016). Reconstitution of giant mammalian synapses in culture for molecular functional and imaging studies. J. Neurosci. 36, 3600–3610. 10.1523/JNEUROSCI.3869-15.2016 27013688 PMC4804015

[B18] Donowitz M. Ming Tse C. Fuster D. (2013). SLC9/NHE gene family, a plasma membrane and organellar family of Na(+)/H(+) exchangers. Mol. Aspects Med. 34, 236–251. 10.1016/j.mam.2012.05.001 23506868 PMC3724465

[B19] Eriksen J. Chang R. McGregor M. Silm K. Suzuki T. Edwards R. H. (2016). Protons regulate vesicular glutamate transporters through an allosteric mechanism. Neuron 90, 768–780. 10.1016/j.neuron.2016.03.026 27133463 PMC4886649

[B20] Ertunc M. Sara Y. Chung C. Atasoy D. Virmani T. Kavalali E. T. (2007). Fast synaptic vesicle reuse slows the rate of synaptic depression in the CA1 region of hippocampus. J. Neurosci. 27, 341–354. 10.1523/JNEUROSCI.4051-06.2007 17215395 PMC6672081

[B21] Farsi Z. Gowrisankaran S. Krunic M. Rammner B. Woehler A. Lafer E. M.. (2018). Clathrin coat controls synaptic vesicle acidification by blocking vacuolar ATPase activity. eLife 7:e32569. 10.7554/eLife.32569 29652249 PMC5935483

[B22] Farsi Z. Jahn R. Woehler A. (2017). Proton electrochemical gradient: driving and regulating neurotransmitter uptake. Bioessays 39:1600240. 10.1002/bies.201600240 28383767

[B23] Ford M. C. Grothe B. Klug A. (2009). Fenestration of the calyx of Held occurs sequentially along the tonotopic axis, is influenced by afferent activity and facilitates glutamate clearance. J. Comp. Neurol. 514, 92–106. 10.1002/cne.21998 19260071

[B24] Forsythe I. D. (1994). Direct patch recording from identified presynaptic terminals mediating glutamatergic EPSCs in the rat CNS, *in vitro*. J. Physiol. 479, 381–387. 10.1113/jphysiol.1994.sp020303 7837096 PMC1155757

[B25] Fremeau R. T. Jr. Kam K. Qureshi T. Johnson J. Copenhagen D. R. Storm-Mathisen J.. (2004). Vesicular glutamate transporters 1 and 2 target to functionally distinct synaptic release sites. Science 304, 1815–1819. 10.1126/science.1097468 15118123

[B26] Fremeau R. T. Jr. Troyer M. D. Pahner I. Nygaard G. O. Tran C. H. Reimer R. J.. (2001). The expression of vesicular glutamate transporters defines two classes of excitatory synapse. Neuron 31, 247–260. 10.1016/s0896-6273(01)00344-0 11502256

[B27] Fujiyama F. Furuta T. Kaneko T. (2001). Immunocytochemical localization of candidates for vesicular glutamate transporters in the rat cerebral cortex. J. Comp. Neurol. 435, 379–387. 10.1002/cne.1037 11406819

[B28] Futai K. Okada M. Matsuyama K. Takahashi T. (2001). High-fidelity transmission acquired via a developmental decrease in NMDA receptor expression at an auditory synapse. J. Neurosci. 21, 3342–3349. 10.1523/JNEUROSCI.21-10-03342.2001 11331363 PMC6762464

[B29] Gan Q. Watanabe S. (2018). Synaptic vesicle endocytosis in different model systems. Front. Cell Neurosci. 12:171. 10.3389/fncel.2018.00171 30002619 PMC6031744

[B30] Goh G. Y. Huang H. Ullman J. Borre L. Hnasko T. S. Trussell L. O.. (2011). Presynaptic regulation of quantal size: K+/H+ exchange stimulates vesicular glutamate transport. Nat. Neurosci. 14, 1285–1292. 10.1038/nn.2898 21874016 PMC3183113

[B31] Goncalves P. P. Meireles S. M. Neves P. Vale M. G. (1999a). Ionic selectivity of the Ca2+/H+ antiport in synaptic vesicles of sheep brain cortex. Brain Res. Mol. Brain Res. 67, 283–291. 10.1016/s0169-328x(99)00081-9 10216226

[B32] Goncalves P. P. Meireles S. M. Neves P. Vale M. G. (1999b). Synaptic vesicle Ca2+/H+ antiport: dependence on the proton electrochemical gradient. Brain Res. Mol. Brain Res. 71, 178–184. 10.1016/s0169-328x(99)00183-7 10521572

[B33] Gowrisankaran S. Houy S. Del Castillo J. G. P. Steubler V. Gelker M. Kroll J.. (2020). Endophilin-A coordinates priming and fusion of neurosecretory vesicles via intersectin. Nat. Commun. 11:1266. 10.1038/s41467-020-14993-8 32152276 PMC7062783

[B34] Guillaud L. Dimitrov D. Takahashi T. (2017). Presynaptic morphology and vesicular composition determine vesicle dynamics in mouse central synapses. eLife 6:e24845. 10.7554/eLife.24845 28432787 PMC5423771

[B35] Hartinger J. Jahn R. (1993). An anion binding site that regulates the glutamate transporter of synaptic vesicles. J. Biol. Chem. 268, 23122–23127. 8226829

[B36] He H. Mahnke A. H. Doyle S. Fan N. Wang C. C. Hall B. J.. (2012). Neurodevelopmental role for VGLUT2 in pyramidal neuron plasticity, dendritic refinement and in spatial learning. J. Neurosci. 32, 15886–15901. 10.1523/JNEUROSCI.4505-11.2012 23136427 PMC3501834

[B37] Herman M. A. Ackermann F. Trimbuch T. Rosenmund C. (2014). Vesicular glutamate transporter expression level affects synaptic vesicle release probability at hippocampal synapses in culture. J. Neurosci. 34, 11781–11791. 10.1523/JNEUROSCI.1444-14.2014 25164673 PMC6608414

[B38] Herzog E. Takamori S. Jahn R. Brose N. Wojcik S. M. (2006). Synaptic and vesicular co-localization of the glutamate transporters VGLUT1 and VGLUT2 in the mouse hippocampus. J. Neurochem. 99, 1011–1018. 10.1111/j.1471-4159.2006.04144.x 16942593

[B39] Hoffpauir B. K. Grimes J. L. Mathers P. H. Spirou G. A. (2006). Synaptogenesis of the calyx of Held: rapid onset of function and one-to-one morphological innervation. J. Neurosci. 26, 5511–5523. 10.1523/JNEUROSCI.5525-05.2006 16707803 PMC6675295

[B40] Hoffpauir B. K. Kolson D. R. Mathers P. H. Spirou G. A. (2010). Maturation of synaptic partners: functional phenotype and synaptic organization tuned in synchrony. J. Physiol. 588, 4365–4385. 10.1113/jphysiol.2010.198564 20855433 PMC3008845

[B41] Hori T. Takahashi T. (2012). Kinetics of synaptic vesicle refilling with neurotransmitter glutamate. Neuron 76, 511–517. 10.1016/j.neuron.2012.08.013 23141063

[B42] Huang H. Trussell L. O. (2014). Presynaptic HCN channels regulate vesicular glutamate transport. Neuron 84, 340–346. 10.1016/j.neuron.2014.08.046 25263752 PMC4254032

[B43] Ikeda K. Bekkers J. M. (2009). Counting the number of releasable synaptic vesicles in a presynaptic terminal. Proc. Natl. Acad. Sci. U S A 106, 2945–2950. 10.1073/pnas.0811017106 19202060 PMC2650301

[B44] Ishikawa T. Sahara Y. Takahashi T. (2002). A single packet of transmitter does not saturate postsynaptic glutamate receptors. Neuron 34, 613–621. 10.1016/s0896-6273(02)00692-x 12062044

[B45] Iwasaki S. Takahashi T. (1998). Developmental changes in calcium channel types mediating synaptic transmission in rat auditory brainstem. J. Physiol. 509, 419–423. 10.1111/j.1469-7793.1998.419bn.x 9575291 PMC2230976

[B46] Iwasaki S. Takahashi T. (2001). Developmental regulation of transmitter release at the calyx of held in rat auditory brainstem. J. Physiol. 534, 861–871. 10.1111/j.1469-7793.2001.00861.x 11483715 PMC2278747

[B47] Juge N. Gray J. A. Omote H. Miyaji T. Inoue T. Hara C.. (2010). Metabolic control of vesicular glutamate transport and release. Neuron 68, 99–112. 10.1016/j.neuron.2010.09.002 20920794 PMC2978156

[B48] Juge N. Yoshida Y. Yatsushiro S. Omote H. Moriyama Y. (2006). Vesicular glutamate transporter contains two independent transport machineries. J. Biol. Chem. 281, 39499–39506. 10.1074/jbc.M607670200 17046815

[B49] Kaila K. Price T. J. Payne J. A. Puskarjov M. Voipio J. (2014). Cation-chloride cotransporters in neuronal development, plasticity and disease. Nat. Rev. Neurosci. 15, 637–654. 10.1038/nrn3819 25234263 PMC4294553

[B50] Kandler K. Friauf E. (1993). Pre- and postnatal development of efferent connections of the cochlear nucleus in the rat. J. Comp. Neurol. 328, 161–184. 10.1002/cne.903280202 8423239

[B51] Kaneko T. Fujiyama F. (2002). Complementary distribution of vesicular glutamate transporters in the central nervous system. Neurosci. Res. 42, 243–250. 10.1016/s0168-0102(02)00009-3 11985876

[B52] Kondapalli K. C. Prasad H. Rao R. (2014). An inside job: how endosomal Na(+)/H(+) exchangers link to autism and neurological disease. Front. Cell Neurosci. 8:172. 10.3389/fncel.2014.00172 25002837 PMC4066934

[B53] Kroll J. Jaime Tobon L. M. Vogl C. Neef J. Kondratiuk I. Konig M.. (2019). Endophilin-A regulates presynaptic Ca(2+) influx and synaptic vesicle recycling in auditory hair cells. EMBO J. 38:e100116. 10.15252/embj.2018100116 30733243 PMC6396150

[B54] Lee U. Choi C. Ryu S. H. Park D. Lee S. E. Kim K.. (2021a). SCAMP5 plays a critical role in axonal trafficking and synaptic localization of NHE6 to adjust quantal size at glutamatergic synapses. Proc. Natl. Acad. Sci. U S A 118:e2011371118. 10.1073/pnas.2011371118 33372133 PMC7812776

[B55] Lee U. Ryu S. H. Chang S. (2021b). SCAMP5 mediates activity-dependent enhancement of NHE6 recruitment to synaptic vesicles during synaptic plasticity. Mol. Brain 14:47. 10.1186/s13041-021-00763-0 33663553 PMC7934559

[B56] Li H. Santos M.S. Park C.K. Dobry Y. Voglmaier S.M. (2017). VGLUT2 trafficking is differentially regulated by adaptor proteins AP-1 and AP-3. Front. Cell Neurosci. 11:324. 10.3389/fncel.2017.00324 29123471 PMC5662623

[B57] Martineau M. Guzman R. E. Fahlke C. Klingauf J. (2017). VGLUT1 functions as a glutamate/proton exchanger with chloride channel activity in hippocampal glutamatergic synapses. Nat. Commun. 8:2279. 10.1038/s41467-017-02367-6 29273736 PMC5741633

[B58] Maycox P. R. Deckwerth T. Hell J. W. Jahn R. (1988). Glutamate uptake by brain synaptic vesicles. energy dependence of transport and functional reconstitution in proteoliposomes. J. Biol. Chem. 263, 15423–15428. 2902091

[B59] Moechars D. Weston M. C. Leo S. Callaerts-Vegh Z. Goris I. Daneels G.. (2006). Vesicular glutamate transporter VGLUT2 expression levels control quantal size and neuropathic pain. J. Neurosci. 26, 12055–12066. 10.1523/JNEUROSCI.2556-06.2006 17108179 PMC6674853

[B60] Morrow E. M. Yoo S. Y. Flavell S. W. Kim T. K. Lin Y. Hill R. S.. (2008). Identifying autism loci and genes by tracing recent shared ancestry. Science 321, 218–223. 10.1126/science.1157657 18621663 PMC2586171

[B61] Naito S. Ueda T. (1985). Characterization of glutamate uptake into synaptic vesicles. J. Neurochem. 44, 99–109. 10.1111/j.1471-4159.1985.tb07118.x 2856886

[B62] Nakakubo Y. Abe S. Yoshida T. Takami C. Isa M. Wojcik S. M.. (2020). Vesicular glutamate transporter expression ensures high-fidelity synaptic transmission at the calyx of held synapses. Cell Rep. 32:108040. 10.1016/j.celrep.2020.108040 32814044

[B63] Neher E. (2010). What is rate-limiting during sustained synaptic activity: vesicle supply or the availability of release sites. Front. Synaptic. Neurosci. 2:144. 10.3389/fnsyn.2010.00144 21423530 PMC3059671

[B64] Ni B. Rosteck P. R. Jr. Nadi N. S. Paul S. M. (1994). Cloning and expression of a cDNA encoding a brain-specific Na(+)-dependent inorganic phosphate cotransporter. Proc. Natl. Acad. Sci. U S A 91, 5607–5611. 10.1073/pnas.91.12.5607 8202535 PMC44045

[B65] O’Donovan S.M. Sullivan C.R. McCullumsmith R.E. (2017). The role of glutamate transporters in the pathophysiology of neuropsychiatric disorders. NPJ Schizophr. 3:32. 10.1038/s41537-017-0037-1 28935880 PMC5608761

[B66] Ono Y. Mori Y. Egashira Y. Sumiyama K. Takamori S. (2019). Expression of plasma membrane calcium ATPases confers Ca(2+)/H(+) exchange in rodent synaptic vesicles. Sci. Rep. 9:4289. 10.1038/s41598-019-40557-y 30862855 PMC6414521

[B67] Ozkan E. D. Lee F. S. Ueda T. (1997). A protein factor that inhibits ATP-dependent glutamate and gamma-aminobutyric acid accumulation into synaptic vesicles: purification and initial characterization. Proc. Natl. Acad. Sci. U S A 94, 4137–4142. 10.1073/pnas.94.8.4137 9108118 PMC20581

[B68] Pietrancosta N. Djibo M. Daumas S. El Mestikawy S. Erickson J. D. (2020). Molecular, structural, functional and pharmacological sites for vesicular glutamate transporter regulation. Mol. Neurobiol. 57, 3118–3142. 10.1007/s12035-020-01912-7 32474835 PMC7261050

[B69] Preobraschenski J. Cheret C. Ganzella M. Zander J. F. Richter K. Schenck S.. (2018). Dual and direction-selective mechanisms of phosphate transport by the vesicular glutamate transporter. Cell Rep. 23, 535–545. 10.1016/j.celrep.2018.03.055 29642010

[B70] Preobraschenski J. Zander J. F. Suzuki T. Ahnert-Hilger G. Jahn R. (2014). Vesicular glutamate transporters use flexible anion and cation binding sites for efficient accumulation of neurotransmitter. Neuron 84, 1287–1301. 10.1016/j.neuron.2014.11.008 25433636

[B71] Price G. D. Trussell L. O. (2006). Estimate of the chloride concentration in a central glutamatergic terminal: a gramicidin perforated-patch study on the calyx of Held. J. Neurosci. 26, 11432–11436. 10.1523/JNEUROSCI.1660-06.2006 17079672 PMC6674540

[B72] Ramet L. Zimmermann J. Bersot T. Poirel O. De Gois S. Silm K.. (2017). Characterization of a human point mutation of VGLUT3 (p.A211V) in the rodent brain suggests a nonuniform distribution of the transporter in synaptic vesicles. J. Neurosci. 37, 4181–4199. 10.1523/JNEUROSCI.0282-16.2017 28314816 PMC6596587

[B73] Reimer R. J. (2013). SLC17: a functionally diverse family of organic anion transporters. Mol. Aspects Med. 34, 350–359. 10.1016/j.mam.2012.05.004 23506876 PMC3927456

[B74] Rizzoli S. O. Betz W. J. (2005). Synaptic vesicle pools. Nat. Rev. Neurosci. 6, 57–69. 10.1038/nrn1583 15611727

[B75] Sakaba T. Neher E. (2001). Calmodulin mediates rapid recruitment of fast-releasing synaptic vesicles at a calyx-type synapse. Neuron 32, 1119–1131. 10.1016/s0896-6273(01)00543-8 11754842

[B76] Satzler K. Sohl L. F. Bollmann J. H. Borst J. G. Frotscher M. Sakmann B.. (2002). Three-dimensional reconstruction of a calyx of Held and its postsynaptic principal neuron in the medial nucleus of the trapezoid body. J. Neurosci. 22, 10567–10579. 10.1523/JNEUROSCI.22-24-10567.2002 12486149 PMC6758464

[B77] Schenck S. Wojcik S. M. Brose N. Takamori S. (2009). A chloride conductance in VGLUT1 underlies maximal glutamate loading into synaptic vesicles. Nat. Neurosci. 12, 156–162. 10.1038/nn.2248 19169251

[B78] Schneggenburger R. Forsythe I. D. (2006). The calyx of Held. Cell Tissue Res. 326, 311–337. 10.1007/s00441-006-0272-7 16896951

[B79] Schuske K. Jorgensen E. M. (2004). Neuroscience. vesicular glutamate transporter—shooting blanks. Science 304, 1750–1752. 10.1126/science.1100475 15205517

[B80] Schwede M. Garbett K. Mirnics K. Geschwind D. H. Morrow E. M. (2014). Genes for endosomal NHE6 and NHE9 are misregulated in autism brains. Mol. Psychiatry 19, 277–279. 10.1038/mp.2013.28 23508127 PMC3932404

[B81] Siksou L. Silm K. Biesemann C. Nehring R. B. Wojcik S. M. Triller A.. (2013). A role for vesicular glutamate transporter 1 in synaptic vesicle clustering and mobility. Eur. J. Neurosci. 37, 1631–1642. 10.1111/ejn.12199 23581566

[B82] Sun J. Y. Wu L. G. (2001). Fast kinetics of exocytosis revealed by simultaneous measurements of presynaptic capacitance and postsynaptic currents at a central synapse. Neuron 30, 171–182. 10.1016/s0896-6273(01)00271-9 11343653

[B83] Tabb J. S. Kish P. E. Van Dyke R. Ueda T. (1992). Glutamate transport into synaptic vesicles. roles of membrane potential, pH gradient and intravesicular pH. J. Biol. Chem. 267, 15412–15418. 1353494

[B84] Takami C. Eguchi K. Hori T. Takahashi T. (2017). Impact of vesicular glutamate leakage on synaptic transmission at the calyx of Held. J. Physiol. 595, 1263–1271. 10.1113/JP273467 27801501 PMC5309358

[B85] Takamori S. (2006). VGLUTs: “exciting” times for glutamatergic research. Neurosci. Res. 55, 343–351. 10.1016/j.neures.2006.04.016 16765470

[B86] Takamori S. (2016). Presynaptic molecular determinants of quantal size. Front. Synaptic Neurosci. 8:2. 10.3389/fnsyn.2016.00002 26903855 PMC4744840

[B87] Takamori S. Holt M. Stenius K. Lemke E. A. Gronborg M. Riedel D.. (2006). Molecular anatomy of a trafficking organelle. Cell 127, 831–846. 10.1016/j.cell.2006.10.030 17110340

[B88] Takamori S. Rhee J. S. Rosenmund C. Jahn R. (2000). Identification of a vesicular glutamate transporter that defines a glutamatergic phenotype in neurons. Nature 407, 189–194. 10.1038/35025070 11001057

[B89] Taoufiq Z. Ninov M. Villar-Briones A. Wang H. Y. Sasaki T. Roy M. C.. (2020). Hidden proteome of synaptic vesicles in the mammalian brain. Proc. Natl. Acad. Sci. U S A 117, 33586–33596. 10.1073/pnas.2011870117 33376223 PMC7776996

[B90] Trojanova J. Kulik A. Janacek J. Kralikova M. Syka J. Turecek R. (2014). Distribution of glycine receptors on the surface of the mature calyx of Held nerve terminal. Front. Neural Circuits 8:120. 10.3389/fncir.2014.00120 25339867 PMC4186306

[B91] Turecek R. Trussell L. O. (2001). Presynaptic glycine receptors enhance transmitter release at a mammalian central synapse. Nature 411, 587–590. 10.1038/35079084 11385573

[B92] Turecek R. Trussell L. O. (2002). Reciprocal developmental regulation of presynaptic ionotropic receptors. Proc. Natl. Acad. Sci. U S A 99, 13884–13889. 10.1073/pnas.212419699 12370408 PMC129792

[B93] Vinatier J. Herzog E. Plamont M. A. Wojcik S. M. Schmidt A. Brose N.. (2006). Interaction between the vesicular glutamate transporter type 1 and endophilin A1, a protein essential for endocytosis. J. Neurochem. 97, 1111–1125. 10.1111/j.1471-4159.2006.03821.x 16606361

[B94] Voglmaier S. M. Kam K. Yang H. Fortin D. L. Hua Z. Nicoll R. A.. (2006). Distinct endocytic pathways control the rate and extent of synaptic vesicle protein recycling. Neuron 51, 71–84. 10.1016/j.neuron.2006.05.027 16815333

[B95] Watanabe S. Rost B. R. Camacho-Perez M. Davis M. W. Sohl-Kielczynski B. Rosenmund C.. (2013). Ultrafast endocytosis at mouse hippocampal synapses. Nature 504, 242–247. 10.1038/nature12809 24305055 PMC3957339

[B96] Watanabe S. Trimbuch T. Camacho-Perez M. Rost B. R. Brokowski B. Sohl-Kielczynski B.. (2014). Clathrin regenerates synaptic vesicles from endosomes. Nature 515, 228–233. 10.1038/nature13846 25296249 PMC4291189

[B97] Weston M. C. Nehring R. B. Wojcik S. M. Rosenmund C. (2011). Interplay between VGLUT isoforms and endophilin A1 regulates neurotransmitter release and short-term plasticity. Neuron 69, 1147–1159. 10.1016/j.neuron.2011.02.002 21435559

[B98] Wilson N. R. Kang J. Hueske E. V. Leung T. Varoqui H. Murnick J. G.. (2005). Presynaptic regulation of quantal size by the vesicular glutamate transporter VGLUT1. J. Neurosci. 25, 6221–6234. 10.1523/JNEUROSCI.3003-04.2005 15987952 PMC6725055

[B99] Winter S. Brunk I. Walther D. J. Holtje M. Jiang M. Peter J. U.. (2005). Galphao2 regulates vesicular glutamate transporter activity by changing its chloride dependence. J. Neurosci. 25, 4672–4680. 10.1523/JNEUROSCI.0549-05.2005 15872115 PMC6725018

[B100] Wojcik S. M. Rhee J. S. Herzog E. Sigler A. Jahn R. Takamori S.. (2004). An essential role for vesicular glutamate transporter 1 (VGLUT1) in postnatal development and control of quantal size. Proc. Natl. Acad. Sci. U S A 101, 7158–7163. 10.1073/pnas.0401764101 15103023 PMC406482

[B101] Wolosker H. de Souza D. O. de Meis L. (1996). Regulation of glutamate transport into synaptic vesicles by chloride and proton gradient. J. Biol. Chem. 271, 11726–11731. 10.1074/jbc.271.20.11726 8662610

[B102] Xie X. S. Crider B. P. Stone D. K. (1989). Isolation and reconstitution of the chloride transporter of clathrin-coated vesicles. J. Biol. Chem. 264, 18870–18873. 2572598

[B103] Yamashita T. Hige T. Takahashi T. (2005). Vesicle endocytosis requires dynamin-dependent GTP hydrolysis at a fast CNS synapse. Science 307, 124–127. 10.1126/science.1103631 15637282

[B104] Zhang X. M. Francois U. Silm K. Angelo M. F. Fernandez-Busch M. V. Maged M.. (2019). A proline-rich motif on VGLUT1 reduces synaptic vesicle super-pool and spontaneous release frequency. eLife 8:e50401. 10.7554/eLife.50401 31663854 PMC6861006

